# Supplementary material for: Methods of In Situ Quantitative Root Biology
Source: Plants (Basel). 2021 Nov 6;10(11):2399. doi: 10.3390/plants10112399 (PMC8625443; doi:10.3390/plants10112399)
Supplement: Supplementary file 1 [file plants-10-02399-s001.zip › plants-1365144-Supplementary.pdf]

Figure S1

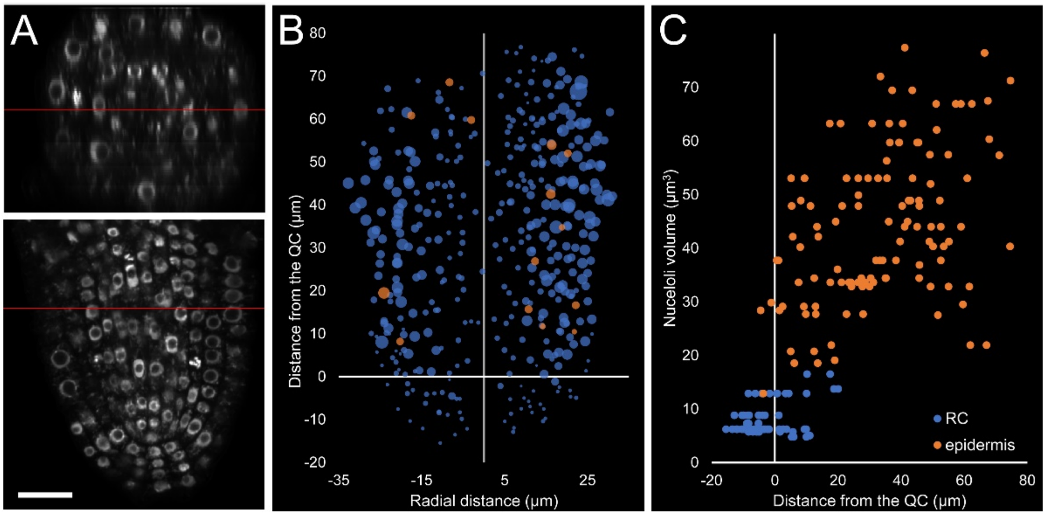

Figure S1. **Nuclei and chromatin analysis in a young Arabidopsis LR**. (A) Confocal 3D image of a young Arabidopsis LR; top: cross section, bottom: longitudinal section. (B). Nucleoli volume distribution along the longitudinal axis: orange indicate mitotic nuclei. (C) Nucleoli volume distribution along the longitudinal axis in different tissues: root cap (RC), blue; and epidermis, orange. Scale bar: 20 µm.

Figure S2

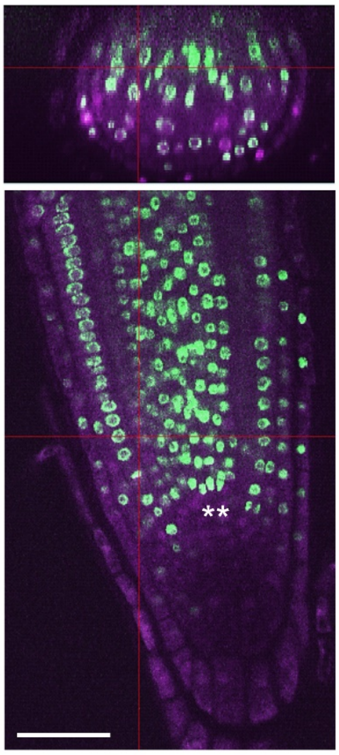

Figure S2. **Long EdU staining of Arabidopsis PR**. Seedlings were incubated with EdU for for 8 h; Confocal 3D image of an Arabidopsis PR; top: cross section, bottom: longitudinal section. QC is marked with asterisks. Scale bar: 50 µm.

Figure S3

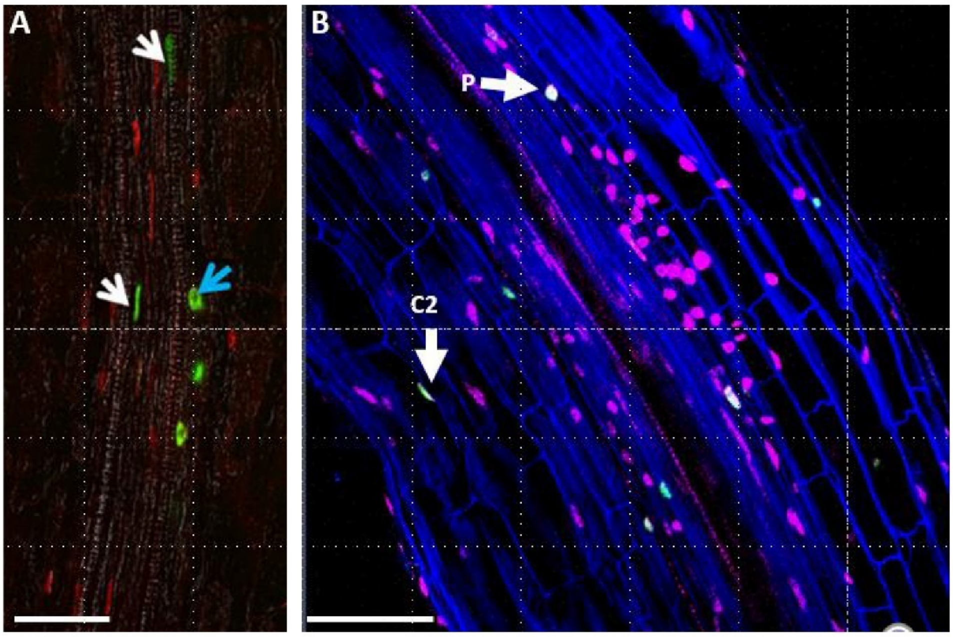

Figure S3. **Analysis of the cell cycle events in the mature part of the plants.** (A) Mature region of an Arabidopsis PR with *de novo* induction of a LR. Red: nuclei staining with DAPI; green: EdU staining; blue arrow: pericycle; white arrow: vascular cells. (B) Mature region of a PR in alfalfa. Blue: cell border staining with calcofluor white; magenta: nuclei staining with PI; green: EdU; C2 and P: DNA replication in cortex 2 and pericycle, respectively. Scale bars: 50 µm.

Figure S4

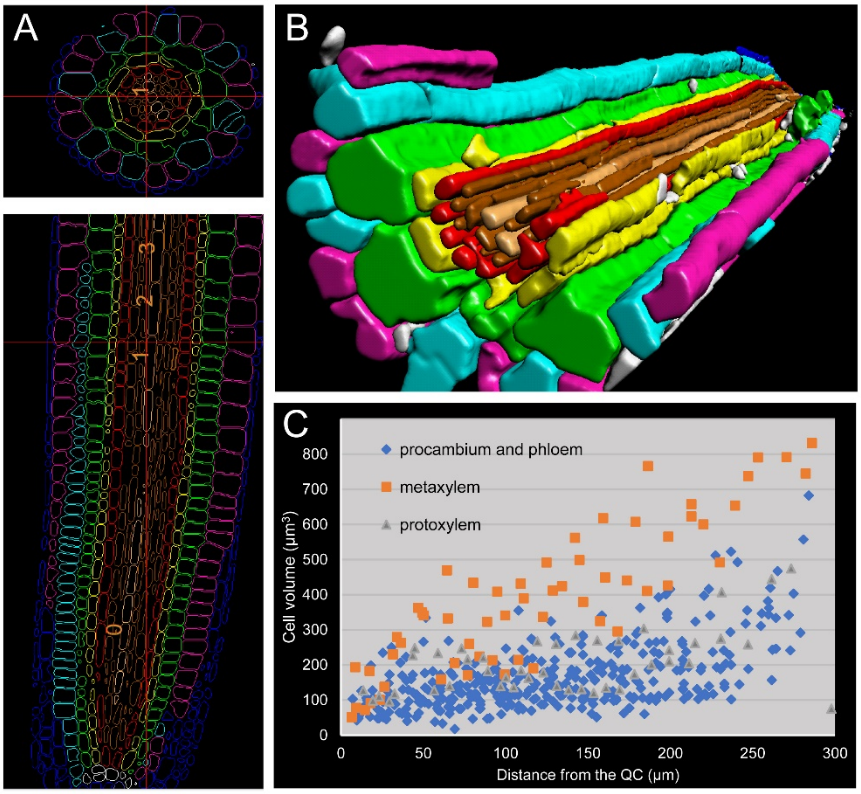

Figure S4. **Vascular analysis in Arabidopsis and foxtail millet PRs.** (A) Virtual 3D image of an Arabidopsis the PR; top: cross section, bottom: longitudinal section. (B) 3D render after segmentation. (C) Cell volume distribution along the longitudinal axis in different tissues: procambium and phloem, blue; metaxylem, orange; protoxylem, grey.

Figure S5

A

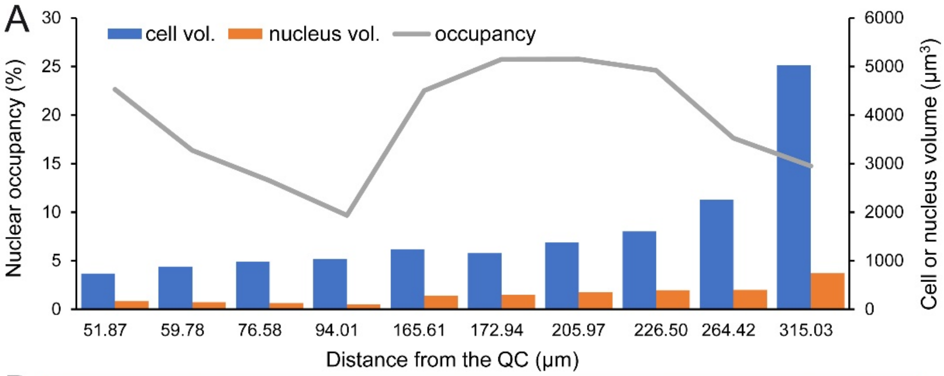

B

Distance from QC	Volume	ESR	Surface Area	Flatness	Elongation	Sphericity
51.87	55.98	3.41	172.00	2.394	2.767	0.612
59.78	63.80	2.48	73.43	2.039	1.693	1.163
64.75	79.80	2.67	80.50	1.865	1.727	1.380
76.58	59.85	2.43	69.84	1.985	1.596	1.189
94.01	100.38	2.88	130.34	1.966	2.365	0.515
165.61	278.49	4.05	197.87	2.061	2.368	1.132
172.94	297.99	4.14	212.39	2.246	2.271	1.048

Figure S5. **Chromatin parameters and nuclear occupancy in RAM and TZ of Arabidopsis atrichoblast cells in the PR.** (A) Cell volume (blue bars); nuclei volume (orange bars) and cell volume/nuclei volume ratio (line). (B) Parameters of individual nuclei extracted by NucleusJ2.0 plugin (Dubos et al., 2020).

Figure S6

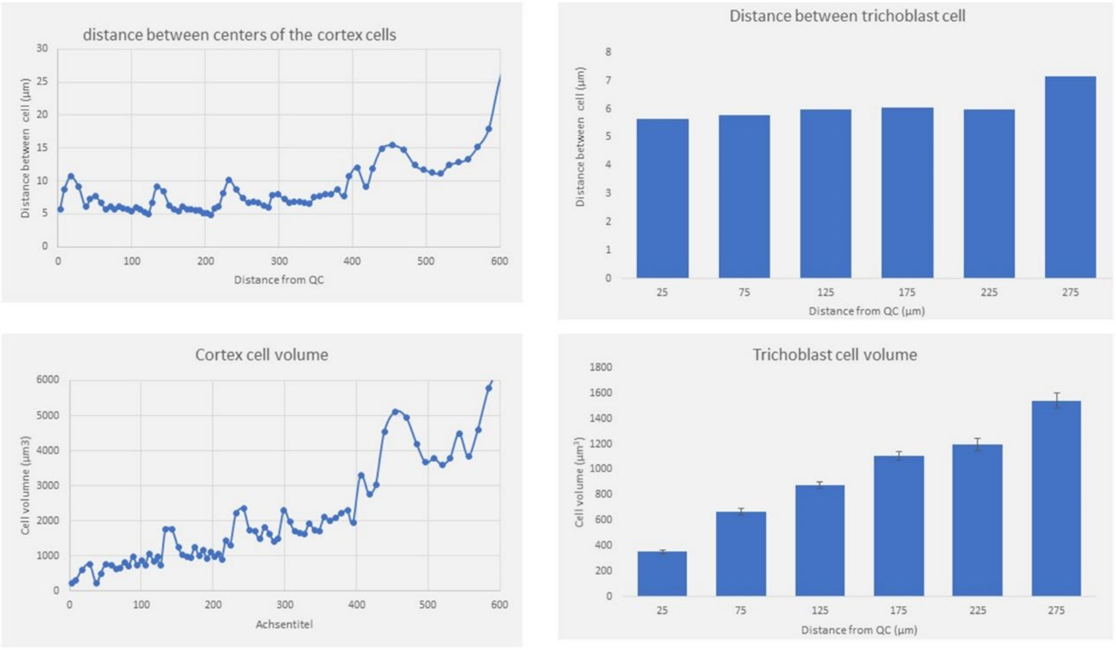

Figure S6. **Length and volume distribution in epidermal cells**. (A) Average distance between cells in the virtual 50 µm sections of the Arabidopsis PR. (B) Average volume in the same sections. In the trichoblast/atrichoblast (and less in cortex) cell volume was continuously increasing; e.g., cell volume of G1 cells at 25 µm is 3 time smaller as cell volume of G1 cells at 200 µm from QC, while cell length remained constant.
